# Relationship between the climbing up and climbing down stairs domain scores on the FES-DMD, the score on the Vignos Scale, age and timed performance of functional activities in boys with Duchenne muscular dystrophy

**DOI:** 10.1590/bjpt-rbf.2014.0063

**Published:** 2014

**Authors:** Lilian A. Y. Fernandes, Fátima A. Caromano, Silvana M. B. Assis, Michele E. Hukuda, Mariana C. Voos, Eduardo V. Carvalho

**Affiliations:** 1 Curso de Fisioterapia, Fonoaudiologia e Terapia Ocupacional, Laboratório de Fisioterapia e Comportamento, Programa de Pós-graduação em Ciências da Reabilitação, Faculdade de Medicina, Universidade de São Paulo (USP), São Paulo, SP, Brazil; 2 Programa de Pós-graduação em Distúrbios do Movimento, Universidade Presbiteriana Mackenzie, São Paulo, SP, Brazil

## Abstract

**BACKGROUND:**

Knowing the potential for and limitations of information generated using different evaluation instruments favors the development of more accurate functional diagnoses and therapeutic decision-making.

**OBJECTIVE:**

To investigate the relationship between the number of compensatory movements when climbing up and going down stairs, age, functional classification and time taken to perform a tested activity (TA) of going up and down stairs in boys with Duchenne muscular dystrophy (DMD).

**METHOD:**

A bank of movies featuring 30 boys with DMD performing functional activities was evaluated. Compensatory movements were assessed using the climbing up and going down stairs domain of the Functional Evaluation Scale for Duchenne Muscular Dystrophy (FES-DMD); age in years; functional classification using the Vignos Scale (VS), and TA using a timer. Statistical analyses were performed using the Spearman correlation test.

**RESULTS:**

There is a moderate relationship between the climbing up stairs domain of the FES-DMD and age (r=0.53, p=0.004) and strong relationships with VS (r=0.72, p=0.001) and TA for this task (r=0.83, p<0.001). There were weak relationships between the going down stairs domain of the FES-DMD-going down stairs with age (r=0.40, p=0.032), VS (r=0.65, p=0.002) and TA for this task (r=0.40, p=0.034).

**CONCLUSION:**

These findings indicate that the evaluation of compensatory movements used when climbing up stairs can provide more relevant information about the evolution of the disease, although the activity of going down stairs should be investigated, with the aim of enriching guidance and strengthening accident prevention. Data from the FES-DMD, age, VS and TA can be used in a complementary way to formulate functional diagnoses. Longitudinal studies and with broader age groups may supplement this information.

## Introduction

Duchenne muscular dystrophy (DMD) is a genetic disease caused by the alteration of gene Xp21, which encodes the protein dystrophin. DMD affects one in every 3,500 male live births^1^. From the clinical point of view, DMD is characterized by progressive, generalized and irreversible muscle weakness, which progresses distally, bilaterally, symmetrically and in an ascending direction. The disease progression is associated with loss of motor skills, mainly in the lower limbs with patients losing their ambulation capacity by age nine to 13 years old. Affected individuals require non-invasive mechanical ventilation from the second decade of life onwards, and death is often due to cardiorespiratory complications^2^^-^ 4.

The decline of the motor activities over the course of the disease is unavoidable. The use of functional assessment scales is needed for clinical follow-up, establishment of the functional diagnosis, and therapeutic decision-making^5^^-^ 7.

There are several functional scales specific for the assessment of individuals with neuromuscular diseases. The Vignos scale (VS)^8^ allows staging of the disease and focuses on functional activities mainly involving the lower limbs, which are considered milestones in the progression of disease. In VS, function is scored from 0 to 10, with higher scores representing poorer functional performance. This classic scale was the basis for the formulation of other scales that aimed at performing a more detailed assessment of the functional capacity of individuals with neuromuscular diseases, such as the Motor Function Measure Scale^9^ (MFM). The MFM assesses functional activities in three dimensions, such as standing and transfers, axial and proximal motor function, and distal motor function, and establishes whether the subject partially completes the exercise, completes it with compensatory movements, or does not perform it at all. The MFM showed good responsiveness, especially in patients with DMD, and was in agreement with the patients' and physicians' perceived changes^10^. The MFM-short form, for use in children aged two to seven years old, showed satisfactory intra- and interexaminer reliability^11^.

On the North Star Ambulatory Assessment scale^12^, activities are graded 0 to 3, depending on whether subjects do or do not perform the activities and whether they perform them in an adapted manner. Formulated for ambulant children with DMD, according to Mazzone et al.^13^, this scale should be used in combination with outcome measures, such as the six-minute walk test, to provide information on different aspects of motor function that may not be captured using a single measure.

The abovementioned scales provide descriptions of compensatory strategies in the performance of tasks but do not afford kinesiological details relative to compensatory movements involving the trunk, pelvis, knees, ankles and feet. They also do not provide detailed information on the movements involved in climbing up or going down stairs, which, according to Vignos, are two of the activities of daily life that should be investigated^8^. One further assessment frequently applied to individuals with DMD is the measurement of time spent in the performance of functional activities, such as climbing up stairs, standing from a seat and walking. Generally, the use of compensatory movements tends to increase the time spent in the performance of the activities tested and thus is indicative of worsening of the functional status^5^^-^ 12.

Observation of functional activities is a simple and accessible ay to assess patients in clinical practice. In addition, such observational tests may be filmed, thus providing a permanent record. Systematized observational analysis is a focus of interest of the authors of the present study, who formulated a specific scale for this purpose: the Functional Evaluation Scale for DMD (FES-DMD). This scale aims at elucidating the potentialities and limitations of the information afforded by systematized observation of functional activities and thus contributes to improving the precision of functional diagnoses and therapeutic decision-making^14^.

The FES-DMD climbing up and going down stairs domain was formulated to allow for specific evaluation of these activities based on systematized observation by means of filming, thus allowing for descriptive analyses of the movements involved, including the compensatory ones, generation of numerical scores, and calculation of the time spent in the performance of the tested activity (TA). The intra- and interexaminer reliability of the FES-DMD has been demonstrated in a previous study^14^.

The TA variable and the VS score are frequently used to classify the functional status of patients. The patients' age also provides an approximation of the clinical progression status^15^. However, few studies have sought to correlate these variables with the presence of compensatory movements in the performance of functional activities^14^^,^ 18, and the contribution of each needs to be understood.

Jung et al*.* 18 assessed the correlation between existing evaluation tools and clinical information on DMD patients, i.e. the Brooke scale, VS, bilateral shoulder abductor and knee extensor muscle power, passive ranges of motion (PROM) of ankle dorsiflexion, angle of scoliosis, peak cough flow (PCF), age, functional muscular shortening (FS), genetic abnormalities and the use of steroids). The results revealed that the scores in the Brooke and Vignos scales increased linearly with age, while the PROM of ankle dorsiflexion exhibited a linear decrease. The muscle power, Cobb angle, PCF and FS varied in their degrees, irrespective of age. Statistically, the genetic abnormalities and use of steroids were not associated with the scores on the functional scales. These findings clearly showed that age should not be used alone.

We believe that the existing scales contribute to the stock of assessment parameters, although it is clear that evaluation routines should be established to monitor the development of each child, adolescent or adult with DMD on an individual basis. Tests and exams that are able to elucidate specific clinical and functional intercurrent events should be used in association at each stage of disease progression.

The objective of this study was to investigate the possible relationships between the number of compensatory movements during climbing up and going down stairs, the functional classification, patients' age and the time required to perform these two tasks in children with DMD.

## Method

### Participants

This study used a film library of 30 children diagnosed with DMD, mean age 7.1 years old (SD: 2.2), mean body weight 40.8 Kg (SD:10.4) and mean height 1.39 m (SD:0.17), made available by Laboratory of Myopathies, Institute of Biosciences, Universidade de São Paulo (USP), São Paulo, SP, Brazil. In the films, the children were performing several functional tasks according to a pre-established script and standardization. The films were made after the children gave informed voluntary consent and their legal guardians signed an informed consent form.

The films included in this study represented children with diagnoses of DMD confirmed by means of DNA testing who were able to climb up and go down stairs without assistance from another person. Further inclusion criteria were as follows: involved in physical therapy at least once per week and using corticoids for at least one year. The films of children requiring lower limb orthoses to perform the investigated tasks were excluded.

The study was conducted at the Laboratory of Physical Therapy and Behavior, Course of Physical Therapy, Faculdade de Medicina (FM), USP, following approval by the research ethics committee of FM/USP (837/05).

### Equipment

The films were recorded with a digital video camera (Digital Camcorder Full HD Sony HDR-CX220 8.9 MP 32x optical zoom) placed on a 1-meter-tall tripod, 3 meters away from and perpendicular to the stairs. The children were filmed in the sagittal plane while performing the indicated tasks, as recommended by FES-DMD. The stairs had six steps to climb up (10 x 27 x 30 cm), four steps to climb down (17 x 25 x 30 cm), being the measure of height, depth and width of each step, respectively, and bilateral standard handrails. Filming started when the investigator gave a verbal command to the child to perform the task as fast as he could.

### Procedure

### Instruments, measurement and data collection

Assessment of functional activity while climbing up and going down stairs was performed using the FES-DMD, a functional scale specific for the functional assessment of children with DMD. This scale assesses activities such as standing from and sitting on a chair and on the ground, climbing up and going down stairs, and walking. The FES-DMD records and describes the movements performed by subjects, with particular focus on compensatory movements. The FES-DMD - climbing up stairs assessment is divided into five phases: preparation/standing, propulsion, swinging, lower limb swinging and stance. The going down stairs assessment is divided into four phases: preparation/standing, propulsion, swinging and stance. In the FES-DMD domain climbing up and going down stairs, the lower the final score, the lower the number of compensatory movements and the better the subject's performance in that activity^14^^,^ 16 ^,^ 18. The indicated task consisted of climbing up six steps and going down four steps to come back to ground level. Prior to filming, the children were requested to sit for five minutes before the performance of the indicated functional tasks so that fatigue would not to impair their performance. Next, the activities of the FES-DMD were requested. The use of aids was allowed.

The films were analyzed by a physical therapist with at least five years of clinical experience in neuropediatrics who was previously trained in the use of the FES-DMD and who was blinded to the study objectives.

To measure the TA, the children were requested to climb the up and go down the stairs as fast as they could. The time elapsed from the moment the investigator gave the verbal command to start to the moment the child's two feet contacted the last stair step was measured. The VS scores and age were extracted from the children's clinical records. VS consisted of 10 functional items with decreasing level of difficulty; the higher the score, the poorer the subject's motor performance^8^.

### Data analysis

The statistical analysis of the data was performed using the software Statistica 11.0 (Statsoft SOUTH AMERICA, 2013). Spearman's correlation test was used to investigate possible associations between FES-DMD-climbing up and going down stairs domain, VS, TA and age. The participants' ages were collected in months and then converted to years through cross-multiplication. The significance level was established as p<0.05.

## Results

The mean score on the FES-DMD-climbing up stairs was 16.7 (SD=8.4), and the mean score on the FES-DMD-going down stairs was 16.0 (SD=8.6). The mean classification score on the VS was 3.1 (SD=1.1). The TA for climbing up the stairs was 11.3 seconds (SD=10.7), and the mean TA for going down the stairs was 11.1 seconds (SD=13.5).

**Figure 1:**
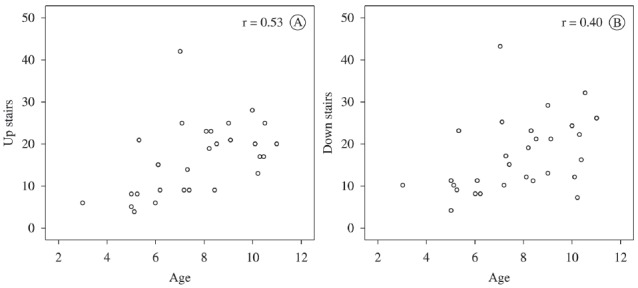
Correlations between the scores on FES-DMD-climbing up the stairs (A) and the scores on FES-DMD-going down the stairs (B) with age (years).

Spearman's correlation test revealed a moderate correlation between the FES-DMD scores for climbing up the stairs and age (r=0.53; p=0.004) and a weak correlation between the FES-DMD scores for going down the stairs and age (Figure 1). The older the subjects' ages, the higher the scores in the FES-DMD-climbing up and going down the stairs (r=0.40; p=0.032).

**Figure 2:**
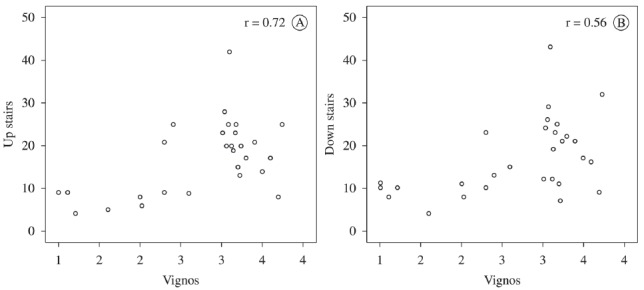
Correlations between the scores on FES-DMD-climbing up the stairs (A) and FES-DMD-going down the stairs (B) with the scores on the Vignos Scale.

Additionally, the VS classification score exhibited correlations with FES-DMD-climbing up and going down the stairs (r=0.72; p<0.001 and r=0.56; p=0.002, respectively) (Figure 2). The worse the functional classification on VS, the higher the number of compensatory movements detected by the FES-DMD on both tasks investigated.

**Figure 3:**
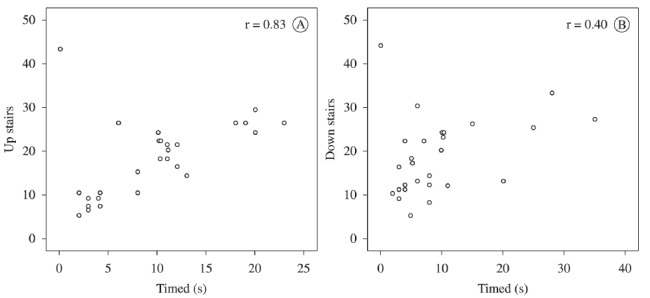
Correlations between the scores on FES-DMD-climbing up the stairs (A) and FES-DMD-going down the stairs (B) with timed performance (seconds).

The FES-DMD-climbing up the stairs was strongly correlated with the TA (r=0.83; p<0.001) (Figure 3), while the correlation between the FES-DMD-going down the stairs and TA was weak (r=0.40; p<0.034) (Figure 3). For both tasks investigated, the higher the TA, the higher the FES-DMD scores, thus indicating a larger number of compensatory movements.

## Discussion

This study investigated the possible relationships between FES-DMD-climbing up and going down the stairs scores with the patients' age and VS classification. In addition, it also investigated the possible relationship of the time taken to do the movement with the activities.

The correlation between the scores on FES-DMD-climbing up and going down the stairs and the subjects' age was moderate to weak. As expected, the older the children's age, the poorer their performance. In a study conducted with 121 children with DMD (mean age=9.9 years old, SD=3.4), Young Jung et al.^18^ found a correlation between the scores on the Brooke and Vignos scales and age. Different from broad-scoped scales, which investigate several tasks as a rule, in the present study, only two highly specific tasks were assessed. For this reason, our results complement the findings reported by those authors.

While individuals with DMD suffer functional losses over time, the wide variability characteristic of the clinical disease progression may make the prognosis difficult to predict, especially when only age is used as the base. Nevertheless, understanding the relationship between age and the clinical course is crucial for understanding the disease progression over time, especially when considering that it may vary widely among the affected individuals.

The main goal of any interventions currently available is to delay the appearance of the complications associated with the disease, modifying its natural course and prolonging the life expectancy of patients. Parreira et al*.* 19 investigated the relationship between age and functional capacity in 90 children with DMD aged five to 12 years old treated with corticoids for approximately seven years. The results did not indicate a significant relationship between age and functional performance, which the authors explained as due to steroid treatment, which delayed the progression of DMD. In an international collaborative study that included 240 individuals with DMD aged two to 28 years old, Henricson et al*.* 20 found that treatment with glucocorticoids preserved clinically meaningful functional milestones and reduced the rate of disease progression as measured by manual muscle testing and other commonly used clinical trial outcome measures, such as pulmonary function tests.

Similar to Parreira et al.^19^, we believe that the reason that the correlation between age and the functional activity of climbing up and going down stairs was moderate to weak in our study was that the children were participating in pharmacological and physical therapy, which may have influenced and minimized the relationship between age and disease progression. In addition, the fact that the sample consisted of children only up to eight years old is relevant in this regard because a larger number of compensatory movements could occur in later stages of disease.

The results evidenced a relationship between FES-DMD-climbing up and going down stairs and the VS. We believe that this finding was favored by the considerable involvement of the lower limbs in the activities tested in the FES-DMD and the VS^8^^,^ 19 ^-^ 21. Further studies are needed to assess the relationship between FES-DMD and other scales.

The correlation between VS and FES-DMD-climbing up stairs was strong, while that with FES-DMD-going down stairs was only moderate. The reason for this difference may be that VS attributes a particular value to the task climbing up stairs because it is considered to be a milestone in the progressive degeneration of the functional capacity of individuals with DMD^8^.

The times spent climbing up and going down the stairs were analyzed and suggested as parameter of assessment in previous studies by Vignos et al.^8^ and Brooke et al.^15^. We found that the FES-DMD-climbing up and going down stairs scores exhibited correlation with the corresponding TA. The time for climbing up the stairs exhibited a stronger correlation with FES-DMD scores compared to the time or going down the stairs (r=0.83 versus r=0.40). The reason for this difference may be the greater motor difficulties involved in climbing up stairs. Although going down stairs demands considerable control of eccentric muscle contraction and coordination, the number of compensatory movements was smaller. We observed that, generally, in the course of going down stairs, the participants tended to accelerate the forward displacement of the center of mass, for which reason the time of movement in this task did not differ much between the groups. This compensatory strategy is compatible with the one reported in the literature^22^. One further factor that accounts for this difference is the greater muscle strength demanded to climb up stairs, which is necessary to support the body's weight against the action of gravity. Upon going down the stairs, the children held the handrail for support, thus losing the fear of falling, with consequent reduction of the time spent on this task.

In the clinical assessment of individuals with DMD, combined use of the TA and the FES-DMD for climbing up and going down stairs is interesting, as although these measures share some components - such as the higher the number of compensatory movements, the longer the time spent in the task - in some cases, the TA may be unchanged as a consequence of an increase in the number of compensatory movements. This finding can only be detected when both assessment tools are used. In such cases, the FES-DMD allowed access to a descriptive characterization of the compensatory movements.

In a similar study, Escorcio et al.^16^ developed the scale FES-DMD-sitting and standing from the ground and demonstrated its reliability. The authors reported a correlation between the scores on the FES-DMD-sitting and standing from the ground and age, VS and TA. Upon investigating the relationship between the FES-DMD scores and age, they found a weak correlation between FES-DMD-sitting on the ground and age but no correlation between FES-DMD-standing from the ground and age. The FES-DMD-sitting on the ground score exhibited a weak correlation with VS (r=0.21), while the FES-DMD-standing from the ground score exhibited a moderate correlation with VS (r=0.56). FES-DMD-sitting on the ground did not have a significant correlation with TA, while the correlation between FES-DMD-standing from the ground and TA was strong (r=0.79). These results are similar to those of the present study, i.e., the correlations between variables were stronger for activities that demanded greater strength and neuromuscular control (i.e., climbing up stairs and standing from the ground).

Hukuda et al.^17^ developed and demonstrated the reliability of the FES-DMD-sitting and standing from a chair. Only the FES-DMD-sitting on the chair score exhibited moderate correlation with age (r=0.44). They also reported moderate correlations between FES-DMD-sitting and standing from a chair with TA and VS (r=0.69 and r=0.66, respectively). VS exhibited few elements in common with the activities sitting and standing from a chair, in contrast to the tasks investigated in the present study, which were also tested in the VS.

Our findings call attention to two facts. The variable age was not adequate when used alone to describe the functional progression of individuals with DMD because it did not reflect the functional alterations that might be observed such tasks as climbing up and going down stairs, which was evidenced by the poor correlation between age and the FES-DMD scores.

In contrast, VS, which is the precursor of the functional scales for assessment of individuals with DMD, showed a strong correlation with FES-DMD-climbing up stairs and a moderate correlation with FES-DMD-going down stairs. Therefore, these two tools could be used in a complementary manner, allowing not only classification but also an understanding of the mechanisms underlying compensatory movements, especially those used to go down stairs.

We believe that the time spent in the performance of the tasks should also be taken into consideration, which was already incorporated into the FES-DMD, as an increase in the number of compensatory movements while climbing up stairs implies a longer time spent in the execution of the task, whereas the time needed to go down the stairs may decrease as a function of abrupt displacements favored by gravity. As we expected, the FES-DMD-climbing up stairs exhibited a strong correlation with the time needed to perform the task.

Our sample showed the relationships existing between the investigated variables relative to children up to approximately eight years old. We believe that future studies could show these relationships in greater detail in groups of patients with different age ranges.

## Conclusion

We conclude that FES-DMD-climbing up stairs bears a moderate correlation with age and strong correlations with VS and the time needed to perform this specific activity. The correlations between FES-DMD-going down stairs and age, VS and the specific TA were weak in children with DMD.

These findings indicate that assessment of the task of climbing up stairs may produce more precise information on the progression of DMD, although the task of going down stairs should also be assessed, with the goal of establishing guidance and accident prevention.

Joint use of the FES-DMD data, age and TA is recommended when making decisions regarding functional diagnoses. Longitudinal studies that include samples with wider age ranges may complement this information.
